# Aneuploidy in human eggs: contributions of the meiotic spindle

**DOI:** 10.1042/BST20200043

**Published:** 2021-01-15

**Authors:** Christopher Thomas, Tommaso Cavazza, Melina Schuh

**Affiliations:** 1 Department of Meiosis, Max Planck Institute for Biophysical Chemistry, 37077 Göttingen, Germany

**Keywords:** aneuploidy, human oocyte, kinetochores, meiosis, spindle

## Abstract

Human eggs frequently contain an incorrect number of chromosomes, a condition termed aneuploidy. Aneuploidy affects ∼10–25% of eggs in women in their early 30s, and more than 50% of eggs from women over 40. Most aneuploid eggs cannot develop to term upon fertilization, making aneuploidy in eggs a leading cause of miscarriages and infertility. The cellular origins of aneuploidy in human eggs are incompletely understood. Aneuploidy arises from chromosome segregation errors during the two meiotic divisions of the oocyte, the progenitor cell of the egg. Chromosome segregation is driven by a microtubule spindle, which captures and separates the paired chromosomes during meiosis I, and sister chromatids during meiosis II. Recent studies reveal that defects in the organization of the acentrosomal meiotic spindle contribute to human egg aneuploidy. The microtubules of the human oocyte spindle are very frequently incorrectly attached to meiotic kinetochores, the multi-protein complexes on chromosomes to which microtubules bind. Multiple features of human oocyte spindles favour incorrect attachments. These include spindle instability and many age-related changes in chromosome and kinetochore architecture. Here, we review how the unusual spindle assembly mechanism in human oocytes contributes to the remarkably high levels of aneuploidy in young human eggs, and how age-related changes in chromosome and kinetochore architecture cause aneuploidy levels to rise even higher as women approach their forties.

## Introduction

Many couples struggle to conceive. Assisted reproductive technologies, such as *in vitro* fertilization (IVF) and intracytoplasmic sperm injections (ICSI) can often alleviate this problem and have experienced a massive boom over the past decades. Every year in the US, ∼1.7% of new-borns are born with the help of assisted reproductive technologies [1]. As part of fertility treatments, human embryos are now routinely observed as they develop — from early after fertilization up to the blastocyst, the stage when the embryo is normally transferred back into the patient for implantation into the uterus. These observations revealed that only ∼50% of fertilized human eggs develop into a blastocyst [2], and of the blastocysts that are transferred back into the patient only ∼50% implant into the uterus [1].

Many human embryos do not develop to term because they have an incorrect number of chromosomes, a condition referred to as aneuploidy [3]. Most embryonic aneuploidy originates from errors during the meiotic divisions in the oocyte or during the first mitotic divisions of the embryo [4]. Mitotic aneuploidy often only affects a subset of blastomeres. Such mosaic human embryos can still develop to term, as the aneuploid cells are often eliminated from the early embryo by mechanisms that are only poorly understood [4]. In contrast, meiotic aneuploidy affects the entire embryo, and most fully aneuploid embryos cannot develop to term [5].

Aneuploidy in human eggs is surprisingly frequent [6]. Already 10–25% of eggs of women in their early thirties are aneuploid, increasing to more than 50% of eggs in women over 40 [7,8]. This makes aneuploidy in eggs a leading cause of pregnancy loss and birth defects in humans [3,9].

Most meiotic aneuploidy results from chromosome segregation errors during the meiotic divisions of the oocyte, the progenitor cell of the egg. Oocyte meiosis is an extremely long process that begins in the female fetus [8]. After the primordial germ cells have reached the genital ridge, they develop into small oocytes that still contain a diploid set of chromosomes. The pairs of homologous chromosomes then become joined via chiasmata in a process termed meiotic recombination. This linked pair of homologous chromosomes is referred to as bivalent [10].

**Figure 1.**
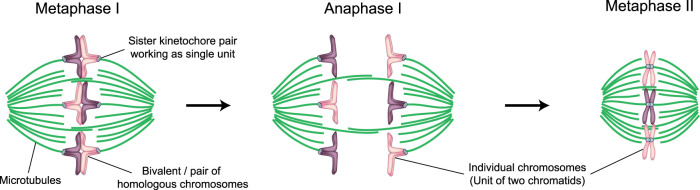
Schematic representation of chromosome organization in human meiosis I and II. (Left) Schematic of the meiosis I metaphase spindle. Green, microtubules. Light blue, kinetochores. Purple and magenta, parental chromosomes. The corresponding maternal and paternal chromosomes are joined by meiotic recombination to form a bivalent. Each bivalent contains two homologous chromosomes, one maternal and one paternal. In meiosis I, the sister kinetochores of each homologous chromosome act as a single unit and are attached to opposite spindle poles. (Middle) Schematic of the meiosis I anaphase spindle. Upon anaphase onset, the bivalent separates into two individual chromosomes, which then move to opposite spindle poles. One set of chromosomes will be eliminated into the first polar body (not represented), while the second set will remain in the egg and become aligned on the metaphase II spindle. (Right) Schematic of the metaphase II spindle. In meiosis II, the sister kinetochores do not act as a single unit anymore, but orient towards opposite spindle poles as in mitosis. Fertilization will trigger anaphase II (not shown), when sister chromatids will be separated. Half of the sister chromatids will be eliminated into the second polar body, while the second half will be enclosed in the maternal pronucleus.

The oocytes subsequently associate with somatic pregranulosa cells to form primordial follicles and arrest in a largely dormant state, referred to as the dictyate stage. Females are generally thought to be born with all of their oocytes already present, meaning that human eggs can be more than 40 years old at the time of fertilization. In puberty, the stored primordial follicles become activated again. The oocyte grows, and in parallel, the somatic cells divide and ultimately form a large Graafian follicle. In the middle of the menstrual cycle, a surge of luteinizing hormone from the pituitary gland causes the oocytes to progress into the first meiotic division [11]. Upon nuclear envelope breakdown (NEBD), a spindle assembles that aligns the bivalents and then eliminates half of the homologous chromosomes into the first polar body. Upon completion of the first meiotic division, there is no reformation of a nucleus or S-phase, but instead, the oocyte progresses directly into the second meiotic division. The remaining homologous chromosomes become aligned on the metaphase II spindle (Figure 1). The oocyte is now referred to as an egg [10]. While the oocyte progresses through the first meiotic division within the follicle, the follicle structure loosens and the egg is eventually ovulated from the ovary into the fallopian tube. The egg then has a narrow time window of up to 24 h during which fertilization must occur [12,13]. Only upon fertilization, the egg progresses into anaphase II and eliminates half of the remaining sister chromatids into the second polar body [10].

Following fertilization, the egg is referred to as a zygote. The maternal and paternal chromosomes become enclosed in two separate pronuclei, which then migrate towards the zygote's center where they break down and subsequently become aligned on the first mitotic spindle. Over the following days, the zygote progresses through a set of rapid mitotic divisions as the embryo migrates through the fallopian tube towards the uterus, where it can eventually implant [10].

As outlined above, embryo development frequently fails, which is often due to high levels of aneuploidy in the eggs that give rise to the embryo. In this review, we summarize recent work that sheds light on how chromosome segregation errors arise within the human oocyte spindle, and how age-related changes in chromosome architecture further exacerbate errors.

## Spindles in human oocytes lack canonical centrosomes and assemble over multiple hours

Chromosome segregation during mitosis is mediated by a spindle that contains two centrosomes at its poles. Canonical centrosomes consist of a pair of centrioles, surrounded by pericentriolar material [14]. They are the major sites of microtubule nucleation in mitotic cells and help to rapidly assemble the spindle upon NEBD.

Centrioles were not detected in human oocyte spindles using either light or electron microscopy [15–18]. The spindles in human oocytes are, therefore, different from somatic cells as they are generally thought to lack canonical centrosomes. In contrast with mouse oocytes, human oocytes also lack prominent acentriolar microtubule organizing centers (aMTOCs) [19,20], which contain many of the components of canonical centrosomes [21], and functionally replace centrosomes in mouse oocytes [22]. Instead of rapidly nucleating microtubules at centrosomes or aMTOCs, human oocytes assemble their spindle through a slow, chromosome-dependent mechanism.

**Figure 2.**
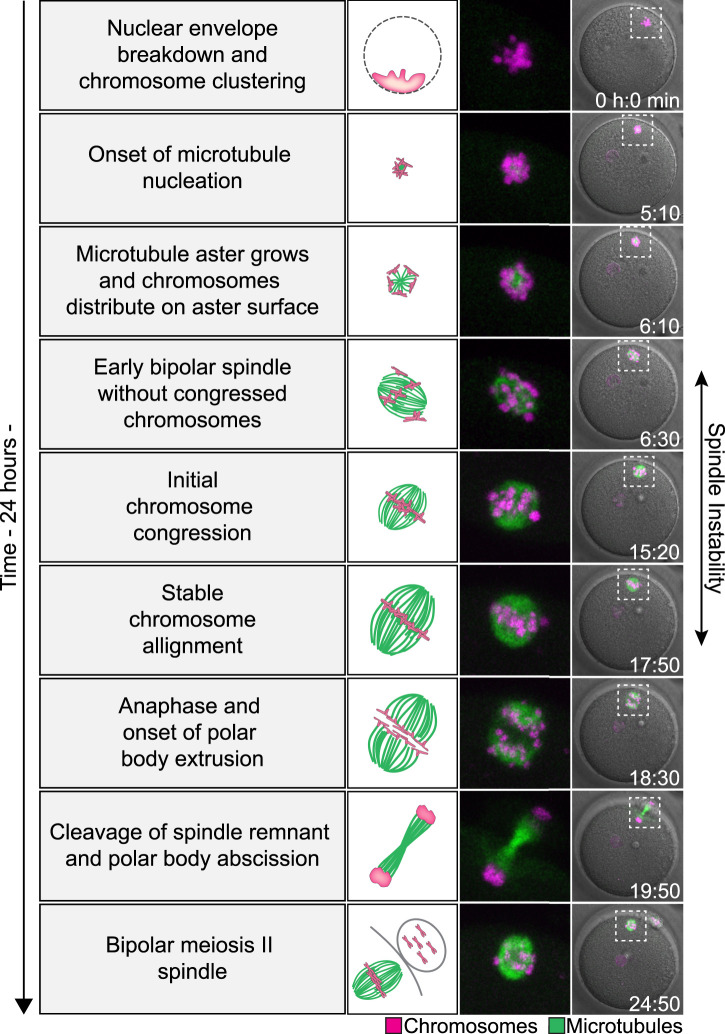
Meiosis in human oocytes. (Left) Schematic of chromatin organization and spindle assembly during human oocyte meiosis. Green, microtubules. Magenta, DNA. (Middle and right). Stills from a representative time-lapse movie of a human oocyte undergoing meiosis. Green, microtubules (EGFP-MAP4). Magenta, DNA (H2B-mRFP1). Merge with differential interference contrast in gray (right). Outlined regions magnified on the side (middle). Time, hours: minutes, 00:00 is nuclear envelope breakdown. Z-projections, 4 sections every 5 μm. Scale bar, 20 μm. Figure adapted with permission from figure 1 in [20].

Upon NEBD, the chromosomes cluster into a single mass. Interestingly, prominent microtubule assembly in human oocytes only starts 4–5 h after NEBD (Figure 2) [20]. Microtubules initially form a small aster within the aggregated chromosomes, and are predominantly associated with kinetochores [20]. As the microtubule aster grows, chromosomes are distributed outward on the aster surface with their kinetochores facing inwards. In humans, the microtubule aster begins to extend into an early bipolar spindle only 6–7 h after NEBD. The spindle poles are initially not well defined, and the chromosomes continuously change their position on the spindle. The chromosomes first align on the metaphase plate ∼13–14 h after NEBD. However, this alignment often does not persist and chromosomes continue to leave the metaphase plate until they stably align ∼16 h after NEBD. Finally, ∼17–18 h after NEBD, the oocyte progresses into anaphase and the homologous chromosomes segregate [20]. Half of the homologous chromosomes are eliminated into the first polar body, while the second half remains in the oocyte. Eventually, ∼20 h after NEBD, the spindle remnant is cleaved and the polar body is abscised. Finally, ∼23 h after NEBD, the oocyte, now referred to as an egg, has assembled the second metaphase spindle and is ready for fertilization (Figure 2) [20].

Altogether, the maturation of an immature human oocyte into an MII arrested egg is an extremely long process, spanning almost 24 h [20,23].

## Spindle assembly in human oocytes relies on Ran-GTP and actin

Spindle assembly in human oocytes is driven by the chromosome-mediated Ran-dependent microtubule assembly pathway [20]. This pathway relies on a gradient of Ran-GTP concentrated around each chromosome. Ran-GTP locally releases spindle assembly factors, such as TPX2 [24], from inhibitory binding to importins and thereby facilitates local microtubule polymerization, and subsequent spindle assembly [24]. Ran-dependent microtubule nucleation was first observed in *Xenopus* egg extracts [25], but later discovered to also be active in somatic cells and human oocytes [20,26]. However, in somatic cells microtubules are also nucleated by the centrosomes, whereas in human oocytes the Ran-GTP pathway is the main source of microtubules. Importantly, the Ran-GTP pathway not only promotes microtubule assembly, but also regulates the activity of several motor proteins. For instance, the Ran-GTP gradient directly controls the activity of HSET, a motor protein important for spindle pole focusing, and Kid, another motor protein necessary for chromosome alignment on the metaphase plate [27,28].

Interestingly, microtubules are not the only structural filaments in the oocyte spindle. Actin filaments are also present and become most prominent from anaphase I onwards [29,30]. Work in mice has shown that actin helps to form the stable microtubule bundles that attach to kinetochores, called kinetochore fibers. Actin is essential in mice for accurate chromosome segregation in meiosis I, and for chromosome congression and segregation in meiosis II [29]. Similarly in human oocytes, actin filaments promote chromosome congression and spindle stability during meiosis II, suggesting a conserved mechanism [30].

## The meiosis I spindle in human oocytes is inherently unstable

**Figure 3.**
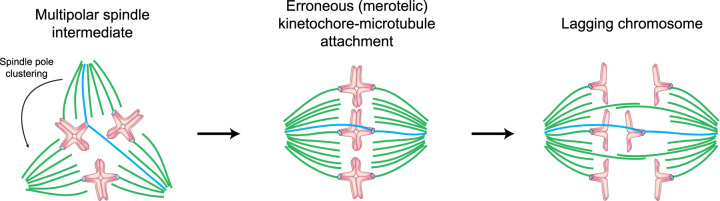
Spindle instability during meiosis I leads to lagging chromosomes in anaphase I. Schematic of a meiosis I spindle progressing through a transient multipolar spindle stage and then undergoing anaphase with lagging chromosomes. Green, microtubules forming correct attachments. Blue, microtubules forming erroneous attachments. Light blue, kinetochores. Magenta, DNA. The meiosis I spindle is often unstable and temporarily multipolar (left). Microtubules become incorrectly attached to kinetochores during the multipolar stages (see microtubules in blue). Error correction is incomplete, resulting in a high number of merotelically attached kinetochores prior to anaphase (middle). Such merotelic attachments are a common cause of lagging chromosomes in anaphase (right). The degree of spindle instability correlates with the degree of lagging chromosomes in anaphase.

During meiosis I in human oocytes, the spindle frequently fails to maintain a bipolar structure after initial bipolarization. Instead, meiosis I spindles often either collapse back into ball-shaped apolar structures or undergo prolonged periods of multipolarity (Figure 3) [20]. During these multipolar stages, kinetochores often become attached to more than one pole. The multiple spindle poles will eventually cluster into two spindle poles and the spindle becomes bipolar again. However, some kinetochores will remain attached to two opposite spindle poles instead of both being attached to a single spindle pole. This attachment error is referred to as merotelic attachment (Figure 3). Interestingly, spindle multipolarity not only favours merotelic attachments in oocytes, but also in cancer cells. Cancer cells often have multiple centrosomes and progress through transient multipolar spindle stages. The multiple centrosomes typically cluster into a bipolar spindle before anaphase onset [31]. However, after centrosome clustering merotelic attachments often persist [32].

In human oocytes, the correction of merotelic attachments before anaphase is often incomplete [20]. Thus, chromosomes frequently remain merotelically attached to the spindle and can lag behind the separating chromosome groups during anaphase (Figure 3). The number of lagging chromosomes correlates with the degree of spindle instability [20]. Consistently, work in mouse oocytes, tissue culture cells, and cancer cells has shown that multipolar spindles favour erroneous kinetochore-microtubule attachments [31–34]. Overall, the frequent occurrence of spindle instability and merotelic attachments in human oocytes provide a potential explanation for why aneuploidy levels in eggs are already high in relatively young women [7,8].

Unlike meiosis I, spindle assembly in meiosis II is relatively fast [20]. Furthermore, multipolarity is infrequent in fixed meiosis II oocytes [30]. Cryopreserved meiosis II oocytes do, however, show spindle defects and may also have unstable spindles [35]. Early studies on fixed non-cryopreserved human MII oocytes made similar observations [36], however, further investigation by live-cell imaging is required to confirm this point.

Moreover, spindle instability together with inefficient chromosome congression is proposed to cause tripolar divisions in meiosis I [37]. Prior to anaphase I onset, chromosomes in human oocytes have occasionally been observed in the spindle as two separated masses. In oocytes where separation occurs, the spindle can undergo a tripolar anaphase, segregating its chromosomes into three distinct groups. After cytokinesis, the chromosomes remaining in the oocyte will reunite to form a single meiosis II spindle carrying an abnormal number of chromosomes [37].

## The spindle assembly checkpoint in human oocytes is not stringent

Erroneous kinetochore-microtubule attachments can result in chromosome congression defects, misaligned chromosomes, and eventually chromosome missegregation and aneuploidy. The spindle assembly checkpoint (SAC) arrests the cell cycle to ensure that all kinetochores are attached to microtubules [38,39]. In tissue culture cells, the SAC can detect a single unattached chromosome and block progression into anaphase [40]. However, in human oocytes, misaligned chromosomes do not block anaphase I onset [20,37] and only severe disruption of the spindle arrests the oocyte in meiosis I [41]. This suggests that human oocytes do not have an efficient SAC in place to block anaphase onset when one or few chromosomes are not congressed and correctly attached to the spindle. SAC signaling in mouse oocytes also lacks stringency. Experiments in which microtubules are partially depolymerized induce only a transient SAC arrest, with oocytes eventually progressing into anaphase regardless of incorrect kinetochore-microtubule attachments [42].

The low stringency of the SAC in human and mouse oocytes is likely due to their large cytoplasmic volume, which in humans is over 200 times that of a dividing somatic cell. Indeed, the SAC was demonstrated to be more efficient in mouse oocytes with smaller cytoplasmic volumes [43]. Another possible explanation proposed for the low stringency of the SAC is that oocytes might have evolved to detect DNA damage rather than chromosome segregation errors, however, this has been excluded in human oocytes [41]. The efficiency of the SAC may also further deteriorate with advancing maternal age as kinetochore localization of the SAC signaling proteins Bub1 and BubR1 is decreased in oocytes from older women [44].

## Human egg aneuploidy is linked to mutations in spindle-related proteins

Recent work identified several mutations of spindle-related proteins that contribute to infertility. One example of this is the TUBB8 gene, which encodes a human and primate-specific isoform of beta tubulin. Beta tubulin forms heterodimers with alpha tubulin and these heterodimers constitute the building blocks for microtubules. Interestingly TUBB8 expression is oocyte-specific [45]. Women with mutations in the TUBB8 gene exhibit a broad spectrum of spindle defects during oocyte meiosis. These range from complete failure in assembling a spindle to severe aneuploidy and arrested embryo development [45,46].

Moreover, genetic analyses have linked infertility to mutant and splice variants of Aurora B and C, two key regulators of kinetochore-microtubule attachment error correction [47–49]. These genetic variants further decrease the accuracy of chromosome segregation in human oocytes and thus increase the rate of aneuploidy.

## Separation of sister kinetochores in meiosis I promotes erroneous microtubule attachments

As we outline above, the spindle in human oocytes is often incorrectly attached to chromosomes, leading to aneuploidy. In the following sections, we describe different types of kinetochore-microtubule attachment defects that have been observed in human oocytes, and discuss how age-related changes in chromosome architecture enhance the formation of incorrect attachments [50–56].

During meiosis I, chromosomes are arranged as a bivalent. The bivalent is composed of two chromosome copies that include two homologous pairs of sister chromatids. Both the homologous chromosomes and sister chromatids within a bivalent are held together by a ring-like structure known as cohesin [57]. Cohesin holding homologous chromosomes together is cleaved in anaphase I. While cohesin holding sister chromatids together is protected in meiosis I, allowing sister chromatids to remain linked until anaphase II.

**Figure 4.**
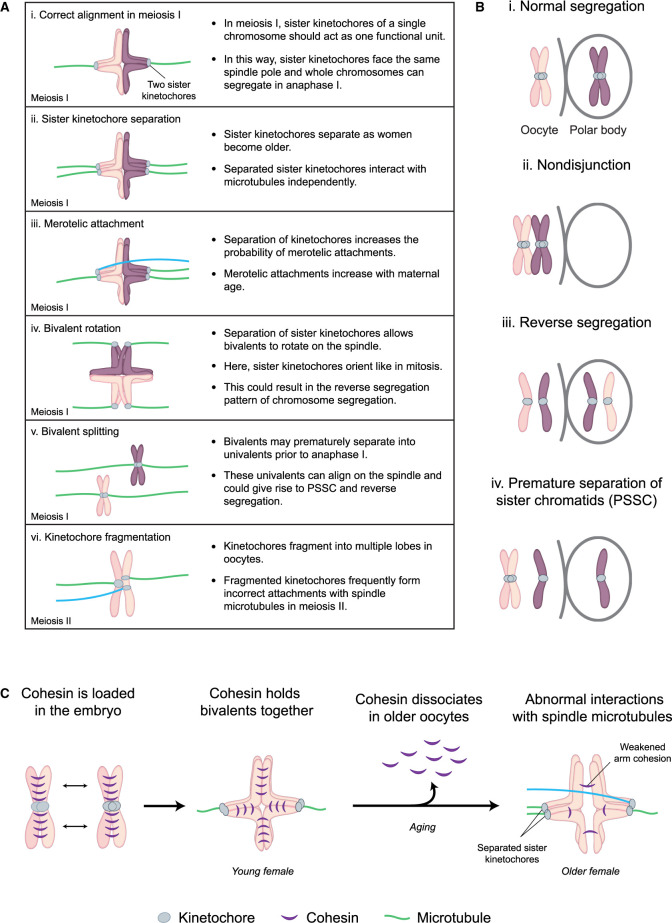
Age-dependent changes in chromosome architecture lead to abnormal attachments with spindle microtubules and segregation errors. (**A**) Schematic showing the ways in which chromosomes abnormally attach to spindle microtubules in human oocytes. Green, microtubules forming correct attachments. Blue, microtubules forming erroneous attachments. Light blue, kinetochores. Purple and magenta, chromosomes. Each bivalent contains two homologous chromosomes, one maternal and one paternal. (i) Sister kinetochores of the same chromosome should act as a single functional unit to allow for segregation of whole chromosomes in anaphase I. (ii) Sister kinetochores frequently separate in oocytes from older women and attach to spindle microtubules independently. (iii) Separation of sister kinetochores increases the probability of merotelic attachments. (iv) Separation of sister kinetochores allows bivalents to rotate on the spindle. Rotated bivalents can become inverted when their sister kinetochores attach to microtubules emanating from opposite spindle poles. (v) Bivalents may prematurely separate into univalents prior to anaphase I. These univalents often align on the metaphase spindle, with their two kinetochores facing in opposite directions and can give rise to both the reverse segregation and PSSC pattern of chromosome segregation in anaphase I. (vi) Kinetochores fragment into multiple lobes in oocytes from older females. The fragmented lobes of these kinetochores can attach independently to spindle microtubules. (**B**). Schematic showing the patterns of chromosome missegregation in human oocytes. Light blue, kinetochores. Purple and magenta, chromosomes. (i) Normal segregation — bivalents are separated in anaphase I into two individual chromosomes. Only one of these is retained within the oocyte and the other is excluded in the polar body. (ii) Nondisjunction — when chromosome segregation fails in anaphase I, leaving the entire bivalent in either the oocyte or polar body. (iii) Reverse segregation - sister chromatids, but not homologous chromosomes, segregate in meiosis I. Consequently, while the oocyte will contain the correct number of chromosomes in meiosis II, the chromatids of the missegregated chromosome will have different parental origins and are not linked. These mismatched chromatids will hence act independently of each other in meiosis II and are, therefore, likely to missegregate during anaphase II. (iv) Premature separation of sister chromatids (PSSC) — where an individual chromatid missegregates during anaphase I. This is a primary cause of aneuploidy in human oocytes. (**C**). Schematic showing cohesin dissociation from chromosomes in oocytes from older women. Green, microtubules forming correct attachments. Blue, microtubules forming erroneous attachments. Light blue, kinetochores. Pink, chromosomes. Purple, cohesin. Cohesin is a ring-like structure that is loaded onto chromosomes in the embryo. Cohesin tethers both homologous chromosomes and sister chromatids together prior to anaphase. In mouse oocytes, cohesin complexes have been shown to gradually dissociate from chromosomes with age. This can result in gaps between homologous chromosomes in oocytes from aged females and an increase in the frequency of abnormal attachments with spindle microtubules.

For entire chromosomes to segregate in anaphase I, the two sister kinetochores of a single chromosome must be fused so that they face the same spindle pole (Figure 1). In this way, two sister kinetochores act as a single functional unit (Figure 4Ai) [58]. However, recent studies observed that sister kinetochores can separate and individually connect to spindle microtubules [54–56]. The separation between sister kinetochores increases dramatically with maternal age in human oocytes [54–56].

When sister kinetochores separate, they may act as two separate functional units and can attach to independent bundles of spindle microtubules (Figure 4Aii). Consequently, separated sister kinetochores are more likely to form attachments to both spindle poles simultaneously (Figure 4Aiii) [54]. These merotelic attachments frequently result in the missegregation of chromosomes during anaphase [8].

Additionally, homologous chromosomes with separated sister kinetochores were observed to rotate on the spindle [54]. Such rotation results in inverted bivalents. This is when in meiosis I, the sister chromatids of each individual homologous chromosome attach to opposite spindle poles — and hence orient like in mitosis — instead of attaching correctly to the same spindle pole (Figure 4Aiv). Inverted bivalents could potentially lead to nondisjunction (Figure 4Bii) or to the reverse segregation of chromosomes [54]. Reverse segregation occurs when sister chromatids, but not homologous chromosomes, segregate in meiosis I (Figure 4Biii) [54,59].

Reverse segregation results in oocytes containing a correct number of chromosomes in meiosis II, though the chromatids are prone to further missegregation as they are no longer linked together by cohesin. These mismatched chromatids will hence act independently of each other in meiosis II and are, therefore, likely to missegregate during anaphase II [54,59]. Alternatively, if one of the two sister chromatid pairs separates in anaphase I, and the other two chromatids stay paired, this leads to the premature separation of sister chromatids (PSSC), where an individual chromatid missegregates during anaphase I (Figure 4Biv) [8]. Indeed, PSSC is a primary cause of aneuploidy in human oocytes [8].

## Premature dissociation of chromosomes and sister chromatids precludes accurate chromosome segregation

In extreme cases, not only can sister kinetochores separate, but also individual sister chromatids can prematurely split apart in meiosis I [54,56,60]. Both the separation of sister kinetochores and sister chromatids are strongly correlated with the amount of cohesin protein present on chromosomes [54,61]. In mouse oocytes, cohesin complexes gradually dissociate from chromosomes during ageing (Figure 4C) [50, 51, 62–65]. This frequently results in large gaps between homologous chromosomes aligned on the metaphase plate of the meiotic spindle in oocytes from aged females [56,60]. These prominent gaps are similarly observed in oocytes from older women [54,56]. However, whether like in mice, this is preceded by a loss of cohesin from chromosomes remains unclear [52,66].

In extreme cases, bivalents prematurely separate into two univalents, prior to anaphase I (Figure 4Av) [54,56,60]. Surprisingly, these univalents often align on the metaphase spindle, with their two kinetochores facing towards opposing spindle poles. When both sister chromatids separate during anaphase I this could give rise to the reverse segregation pattern — the chromosomes would separate like in mitosis instead of separating as would be expected in meiosis I (Figure 4Biii) [8]. Alternatively, if only one chromosome separates into chromatids, and the other one moves to a single spindle pole this would give rise to PSSC (Figure 4Biv) [8]. Both PSSC and reverse segregation correlate with a loss of cohesin from chromosomes with advancing maternal age [8].

Interestingly, a recent study showed that human oocytes in meiosis II contained chromatids that were separated by a large distance, yet were not completely dissociated. Instead, they frequently contained stretched chromatin threads linking the chromatids together [6]. These threads may correspond to regions where residual cohesin is concentrated or potentially point to the existence of alternative connections between chromosomes that may become more important as cohesin levels decrease with advancing maternal age.

## Kinetochore fragmentation correlates with incorrect microtubule attachments

Until now, we have discussed changes within the whole chromosome architecture leading to segregation defects in human oocytes. However, a recent study additionally identified age-related morphological changes of the kinetochore itself [67]. This work showed that chromatin at the centromeres of chromosomes in the oocyte becomes decompacted as females age [67]. Kinetochores assembled at centromeres containing decompacted chromatin frequently lose integrity and fragment into multiple lobes [67].

In meiosis II, these fragmented kinetochore lobes form independent attachments to spindle microtubules (Figure 4Avi). Consistent with this observation, fragmented kinetochores in meiosis II are more likely to attach to kinetochores merotelically [67]. Kinetochore fragmentation was also artificially induced by an acute partial depletion of cohesin in mice. This suggests that compaction of centromeric chromatin and thus kinetochore integrity requires sufficient amounts of cohesin in the centromeric region [67]. Together these data establish that abnormal attachments of chromosomes to the human oocyte spindle can arise both from defects in spindle assembly, as well as from alterations in chromosome and kinetochore architecture that favour incorrect kinetochore-microtubule attachments.

## Summary

Human eggs are frequently aneuploid. This aneuploidy primarily results from chromosome segregation errors during oocyte meiosis. Multiple pathways contribute to the frequency of these errors. One example is the inherent instability of the human meiosis I spindle. Spindle instability favours erroneous kinetochore-microtubule attachments in human oocytes, resulting in errors when chromosomes are segregated during anaphase. Furthermore, the SAC in human oocytes lacks stringency, allowing for progression into anaphase regardless of incorrect kinetochore-microtubule attachments.

In mouse oocytes, cohesin complexes gradually dissociate from chromosomes with age. Cohesin loss is linked to many changes in chromosome architecture that have been shown to contribute to aneuploidy in oocytes. While it is unclear whether human oocytes similarly lose cohesin from their chromosomes with age, separation of sister kinetochores in MI, premature splitting of sister chromatids, and kinetochore fragmentation are all observed with increasing frequency in older human oocytes, suggesting a conserved mechanism.

It is thus clear that multiple factors predispose human oocytes to aneuploidy. A greater understanding of the mechanisms underlying this aneuploidy is required if we are to work towards the development of treatments to counteract this age-related decline in fertility. Until then, however, nature requires that women conceive during a narrow window of fertility.

## Perspectives

- *Highlight the importance of the field*. Human oocytes and embryos are frequently aneuploid, causing pregnancy loss and infertility. Aneuploidy is already high in young women and increases further as women are approaching their forties.
- *A summary of the current thinking*. Spindle instability, multipolarity, and merotelic attachments contribute to high aneuploidy in young and older women. In addition, chromosome and kinetochore architecture change as women get older, leading to a further increase in abnormal interactions between chromosomes and the spindle and a rise in chromosome segregation errors in meiosis I and II.
- *A comment on future directions*. Understanding the causes of high spindle instability in human oocytes and the age-related alterations in chromosome and kinetochore architecture will be crucial for counteracting the maternal age effect in humans.

## Open Access

Open access for this article was enabled by the participation of the Max Planck Institute for Biophysical Chemistry in an all-inclusive *Read & Publish* pilot with Portland Press and the Biochemical Society under a transformative agreement with Max Planck Digital Library.

## Abbreviations

aMTOCs: acentriolar microtubule organizing centers
NEBD: nuclear envelope breakdown
PSSC: premature separation of sister chromatids
SAC: spindle assembly checkpoint
